# Social networks and life satisfaction: The interplay of network density and regulatory focus

**DOI:** 10.1007/s11031-015-9490-1

**Published:** 2015-03-25

**Authors:** Xi Zou, Paul Ingram, E. Tory Higgins

**Affiliations:** 1 London Business School, Regent’s Park, London, NW1 4SA UK; 2 Columbia University, New York, NY USA

**Keywords:** Social networks, Regulatory focus, Life satisfaction, Perceived support

## Abstract

We propose that an individual’s regulatory focus moderates the significant role social network density—the degree of interconnectedness among a person’s social contacts—plays in shaping life satisfaction. Evidence from Study 1 indicates that participants with *high prevention effectiveness* reported *higher* life satisfaction when they were embedded in a *high*-*density* network, whereas participants with *low promotion effectiveness* reported *lower* life satisfaction when they were embedded in a *low*-*density* network. Study 2 further specifies the underlying mechanism, namely that participants with high prevention effectiveness are more likely to obtain support for meeting obligations and responsibilities when they are embedded in a high-density network, whereas participants with low promotion effectiveness suffer from the support for creative inspiration and personal development in a low-density network (by highlighting their promotion failure). Implications for studying the interplay between social networks and individuals’ self-regulatory motives are discussed.

## Introduction

It is undeniable that social relationships matter for individuals’ well-being. Psychologists, sociologists and economists have all reported that individuals who have a larger number of close relationships have a higher level of life satisfaction (e.g., Diener and Seligman 2002; Helliwell and Putnam 2001; Reis and Gable 2003). Social relationships also predict a wide variety of life outcomes that include risk for mental illness, poor physical health, and even death (e.g., Bergmann and Syme 1979; Cohen et al. 1997; House et al. 1988). Although the effect of social relationships is perhaps obvious, a less well documented effect in social psychology is that individuals with networks that are rich with interconnections also reported a higher level of well-being. Using the General Social Survey data, Burt (1984) finds that people with denser networks—a high degree of interconnections among a person’s social contacts—are happier. Indeed, cumulative work from sociology has argued that dense networks facilitate the status-quo maintenance and provide security and stability (Coleman 1990; Granovetter 1985, 1992), which is essential to individuals’ life satisfaction (Baumeister and Leary 1995).

Nevertheless, people may need different kinds of support and resources to live a satisfying life. For example, whereas people are more likely to obtain protective resources and stability from a dense network, such resources may not be valued as much by people who emphasize growth potential and personal achievements. In this regard, the field of social psychology has long argued that human behavior and subjective experience are a function of the interaction between personality and environmental variables (i.e., Funder and Ozer 1983; Kelley 1991; Lewin 1935; Mischel and Shoda 1995). A wealth of evidence has shown that various forms of person-situation fit have important positive consequences for individuals’ psychological well-being (e.g., O’Reilly et al. 1991; Ostroff and Schulte 2007). These findings generalize across different types of fit, including person–job fit, person–organization fit, person–group fit, and person–supervisor fit (e.g., Berson and Halevy 2014; Hoffman and Woehr 2006; Kristof-Brown et al. 2005; Verquer et al. 2003).

The current investigation is in part motivated to identify a new form of person-situation fit: individuals’ regulatory focus orientation and network structure. To be more specific, research on regulatory focus theory has suggested that a satisfying life could be qualitatively different for individuals with a promotion motivation versus a prevention motivation (e.g., Ferris et al. 2013; Grant and Higgins 2003; Higgins et al. 2001). Past studies have shown that success for prevention-focused individuals means maintaining security and the status-quo, but for promotion-focused individuals it means achieving personal ideals and making significant advancements (Higgins 1997; Shah et al. 1998). Such individual differences in regulatory focus give rise to an important question on whether and how individual differences in regulatory focus orientation moderates the effect of network density on life satisfaction^1^ (Busseri and Sadava 2011; Diener 1984; Lucas et al. 1996). That is, the effect of network structure on well-being may depend on how well the structure fits with individuals’ regulatory focus orientation.

In the sections below, we first review the research on social networks and regulatory focus theory, and then specify how networks with either low-level or high-level density could differentially fit with individuals with distinct regulatory focus orientations. We subsequently report two studies that test our predictions. Our goal is to integrate the research on social network density into the psychological research on well-being, as well as to demonstrate the power of individual differences for understanding the effect of network density on well-being.

### Social networks and life satisfaction

Although there is substantial evidence that both the quantity and the quality of social relationships can significantly affect individuals’ well-being, evidence on the effect of the structural characteristics of individuals’ social networks is relatively sparse (see Burt 1984 as an exception). There is research on psychological loneliness that provides some indirect evidence for the link between network density and well-being. In a series of studies, Stokes (1985) sampled individuals’ networks and measured network size, the number of people that individuals feel close to, the percentage of network contacts that are relatives, and network density. Among all the predictors, network density had the strongest and most consistent relationship with loneliness, with denser networks being associated with less loneliness. Stokes evoked the concept of community to explain this effect, suggesting that high-density networks provide individuals with a sense of belonging to a group and a sense of community, which tempers the feeling of loneliness. This alleviation of loneliness has been shown to be essential to psychological well-being (Cacioppo et al. 2003).

Despite the fact that emerging evidence seems to support a positive role of high network density in well-being, a closer look at the full set of evidence for the association between networks and well-being yields a more ambiguous story. A few studies have reported null effects of network density on a specific dimension of well-being, job satisfaction (Brass 1981; Hurlbert 1991). Aside from the mixed evidence, this body of research has suggested that in order to capture the structural characteristics of social network, it is important to examine network density as an entry point that can then serve as a foundation to examine the person-structural fit. We offer a possible resolution of the mixed findings regarding the relation between density and well-being by presenting evidence that the effect of network density on life satisfaction is moderated by individuals’ regulatory focus orientation.

### Promotion versus prevention effectiveness and life satisfaction

We posit that individual differences in self-regulatory concerns are particularly useful for differentiating network density effects on life satisfaction because effective self-regulation is essentially about how individuals successfully use various resources (e.g., psychological, social) to address their primary life concerns. There is already strong evidence that the successful pursuit of personally meaningful goals represents a major source of life satisfaction (e.g., Emmons 1996; Gable 2006). For example, Diener and Fujita (1995) studied the relationship among people’s individual goals, their resources, and their life satisfaction. They found that resources predicted life satisfaction more strongly when they were relevant to an individual’s goals than when they were not.

In pursuing a satisfying life, individuals may differ in their primary life concerns and their preferred end-states. These end states can be conceptualized in terms of different motivational constructs, such as an individual’s motivational dispositions or his or her personal goals (Emmons 1997). A central theory that differentiates these end-states is regulatory focus theory (Higgins 1997). Regulatory focus theory distinguishes between two motivational systems that serve critically important but different basic needs: promotion focus and prevention focus.

The satisfying end-state of a promotion-focused individual is a world filled with the possibility for advancement or gains. What matters for people with a high promotion orientation is to make progress, to move from the current status quo to a better state, to fulfill their hopes and aspirations. Consequently, promotion-focused individuals are concerned with growth and accomplishments and they focus on attaining gains. In contrast, the satisfying end-state of a prevention-focused individual is a world where they can effectively maintain safety and security and meet their responsibilities and obligations. What matters for people with a high prevention orientation is to maintain a satisfactory state by ensuring that bad things or losses do not happen. Consequently, prevention-focused individuals are concerned with safety and responsibility and they focus on maintaining non-losses.

In sum, individuals with a high promotion motivation versus a high prevention motivation need different support to live a satisfying life. The promotion-focused individuals need resources to meet their growth needs and achieve a sense of advancement and accomplishment. In contrast, prevention-focused individuals need protective resources to help them maintain security and stability and meet their responsibilities.

### Regulatory focus and network density

Drawing on this framework, we distinguish two forms of self-regulatory effectiveness—promotion effectiveness and prevention effectiveness, which is widely measured by the individual difference scale of regulatory pride (Higgins et al. 2001). As outlined above, promotion effectiveness is about being effective in reaching ideals and advancement, whereas prevention effectiveness is about being effective in meeting obligations and maintaining security. A high-density network provides prevention-serving support, further facilitating individuals with high prevention effectiveness to maintain a secure status-quo and to address their concerns with meeting their responsibilities. A wealth of research by sociologists have demonstrated that dense network cultivates a coherent set of normative expectations within a collective unit, facilitates reputation control, and is good at maintaining stability and safety (Coleman 1990; Granovetter 1985, 1992). The focus on stability and safety that dense networks facilitate is precisely what matters for individuals with high prevention effectiveness (see Brodscholl et al. 2007; Liberman et al. 1999). That is, the link between prevention-serving support and life satisfaction would be particularly strong among prevention-focused individuals. Hence, we made following hypotheses:

#### **Hypothesis 1a**

Higher network density has a positive effect on life satisfaction among individuals with high prevention effectiveness.

#### **Hypothesis 1b**

Higher density networks are rich in prevention-serving support.

#### **Hypothesis 1c**

Prevention-serving support mediates the effect of a higher density network on life satisfaction among individuals with high prevention effectiveness.

A low-density network is often associated with greater exposure to new information, diversity, and opportunity (Burt 1992, 2005). For example, managers who are connected to contacts who do not know each other (i.e. low density network) tend to be rated higher on creativity performance by their bosses and colleagues (Zou and Ingram 2013). In this regard, research on regulatory focus has shown that individuals with high promotion effectiveness, compared to individuals with low promotion effectiveness, tend to engage in more entrepreneur-related networking activities, value more diverse information, and even uncertain situations (Pollack et al. 2015; Molden and Higgins 2004; Liberman et al. 2001). Individuals with high promotion effectiveness also particularly value opportunities to achieve personal success (Shah and Higgins 2001) and novelty (Friedman and Forster 2001). Thus, a low-density network is more likely to provide promotion-serving support, which fits particularly well with the promotion-focused individuals. Hence, we made following hypotheses:

#### **Hypothesis 2a**

Lower network density has a positive effect on life satisfaction among individuals with high promotion effectiveness.

#### **Hypothesis 2b**

Lower density networks are rich in promotion-serving support.

#### **Hypothesis 2c**

Promotion-serving support mediates the effect of a lower density network on life satisfaction among individuals with high promotion effectiveness.

We tested these hypotheses in two studies. Study 1 used a survey design in which we measured individuals’ social network structures and chronic regulatory focus orientations as a way to provide the initial evidence on the person-structure fit on life satisfaction (Hypotheses 1a and 2a). Study 2 was designed to replicate the findings from Study 1 while further specifying the mediation mechanism by testing the full set of hypotheses (Hypotheses 1a, b, and c, and Hypotheses 2a, b, and c).

## Study 1

### Method

#### Participants and design

We collected the data from four cohorts of managers attending an Executive MBA program at a business school in a large city in the United States. The students were managers who continued full-time work while they studied. A total of 573 managers (26.7 % females) participated in this study. Due to very occasional missing data on some variables, analyses reported below have slightly different sample sizes. Of these, 53 % were Caucasians, 41 % were Asians (Chinese, Japanese, Korean, or Indian), and the rest were of other races (mostly African Americans and Hispanic Americans). Their most common industries of employment were finance and banking (37 %). Typically, the participants held managerial positions in large companies—for example, as vice presidents in international banks, other financial institutions, or consulting firms. Other participants held executive positions in smaller companies (e.g., as CEO of a family business). A smaller group consisted of professionals who had risen to supervisory or managerial positions (e.g., a Ph.D. scientist who led a research project for a large pharmaceutical company). In the first half of their first semester, participants completed an electronic survey about their social networks. Also early in their program, but as part of a different assignment, participants completed an online survey on regulatory focus and life satisfaction. Each participant was given feedback on his or her social-network profile, as well as an individualized report on life satisfaction and regulatory focus orientations as a form of debrief.

##### General life satisfaction

Life satisfaction was measured with three items from the Satisfaction with Life Scale (Diener et al. 1985), consisting of “I am satisfied with my life”, “In most ways my life is close to my ideals”, and “The conditions of my life are excellent.” Prior research has suggested that the first three items form a reliable scale and are better indicators of life satisfaction than the two items that were not used (see Oishi 2004; Schimmack and Oishi 2005). Participants rated these three items on a six-point scale ranging from 1 (*strongly disagree*) to 6 (*strongly agree*). The items were averaged to form the general life satisfaction measure (*M* = 4.74, *SD* = 1.03, α = 0.86).

##### Regulatory focus

Regulatory focus was measured using the Regulatory Focus Questionnaire (RFQ; Higgins et al. 2001), which asks 11 questions, of which the promotion subset (six questions, *M* = 4.08, *SD* = 0.53, α = 0.71) measures individuals’ subjective history of being effective in promotion motivation with questions such as, “How often have you accomplished things that got you ‘psyched’ to work even harder?” The prevention subset (five questions, *M* = 3.58, *SD* = 0.84, α = 0.79) measures individuals’ subjective history of being effective in prevention motivation with items such as, “Not being careful has gotten me into trouble at times” (reverse scored). Thus, this scale captures individual differences in their promotion versus prevention effectiveness. The response scale for these questions ranges from 1 (*never or seldom*) to 5 (*very* *often*).

##### Network survey

The network survey allowed each participant (ego) to list up to 24 contacts (alters) whom they deemed most important for their professional success. The alters could come from any context and were not restricted to the participants’ workplace. For each alter listed, participants were asked to indicate whether the alter worked in the same organization, together with other details on the alters’ background and the nature of their relationship. On average, 46 % of the contacts were from the participants’ workplaces. Clearly, participants felt that many contacts from their “personal” worlds, or at least from outside the workplace, were relevant to their professional networks, and the resulting data therefore seem quite comprehensive. Below are details of the network measures.

##### Tie strength

For each alter, participants answered, “How close do you feel to this person?” by choosing from “Very Close,” “Close,” “Not So Close,” and “Distant.” On average, participants listed 30 % of their alters as very close, 42 % as close, 24 % as not so close, and 4 % as distant. We controlled for the number of strong ties, which was the sum of the number of alters to which each participant felt close and very close. Since a dense network is often associated with a larger number of strong ties, this control helps to specify the effect of network density that is driven by the interconnection among the social network contacts, instead of the tie strength per se.

##### Relationships among alters

After participants had completed the first part of the survey, which involved listing the alters and describing their relationships, the second part of the survey began with the heading, “Who knows whom in your network?” The survey instructions stated, “A positive relationship can be (a) a close relationship (example: when people work very close together or have a high level of friendship) or (b) a positive but not especially close relationship (example: people who know each other but are not in frequent contact, and are not strong friends or enemies).” We also told participants that “negative relationships exist between individuals that dislike each other, and intentionally avoid contact, or even attempt to harm each other.” Negative relationships were rarely reported. When characterizing the alter–alter relationships, we coded the existence of the relationship, regardless of the relationship closeness and valence, “close” and “positive, but not especially close,” as 1, and otherwise coded 0. We also conducted a supplemental analysis that weights the tie strengths by coding the “close” alter relationships as 2 and the “positive, but not especially close” alter relationships as 1. The results were consistent with those we report below, that is one produced using one level of positive ties among alters.

Based on the information of the relationship among alters, we calculated the network density score (Wasserman and Faust 1994). The network density measure is calculated by dividing the total number of identified relationships between the alters by the total possible number of ties, which for an undirected graph is:

$$ \frac{{\sum\nolimits_{{{\text{i}} = 1}}^{\text{g}} {\sum\nolimits_{{{\text{j}} = 1}}^{\text{g}} {{\text{a}}_{\text{ij}} } } }}{{{\text{N}}({\text{N}} - 1)}} $$

where a_ij_ is either 1 or 0, with 1 indicating the existence of a relationship between i and j, and N is the number of nodes in the network.

##### Ego’s demographic background

We controlled for participants’ age, gender, and ethnicity. As noted, Caucasian was the dominant group, and Hispanics, African Americans, and Asians were the minority groups. We included a non-Caucasian indicator variable in our analysis, with the Caucasians forming the omitted category.

##### Ego’s industry background

We obtained descriptions of the participants’ jobs from their biographic entries in the class roster. Given that the largest group of the participants came from the finance industry, we constructed one indicator variable to designate participants who worked in finance.

##### Alters’ demographics

We controlled for other demographic characteristics of the alters collected in the network survey. The survey asked whether each alter was of a different race and/or a different gender than the respondent. Based on this information, we calculated the number of different gender alters and the number of different race alters in each ego’s network as control variables.

### Results

Table 1 shows descriptive statistics and correlations among the key variables in Study 1. Supporting the positive main effect of network density, higher life satisfaction was significantly associated with higher overall network density (r = .15, *p* < .001). Both promotion effectiveness (r = .46, *p* < .001) and prevention effectiveness (r = .15, *p* < .001) showed a significant correlation with life satisfaction, with promotion effectiveness displaying a significantly stronger effect (Pearson-Fillon Z = 5.91, *p* < .001). Consistent with past findings (Helliwell and Putnam 2001), the number of strong ties showed a significant positive relationship with life satisfaction (r = .10, *p* < .002) as well. In this sample, we also observed that female participants reported a higher level of life satisfaction (r = .09, *p* < .03), as did Caucasians (r = .14, *p* < .001). Interestingly, female managers’ overall networks were denser (r = .10, *p* < .035), as were Caucasians’ (r = .12, *p* < .005). There were no significant correlations between network density and the two regulatory focus variables.

**Table 1:** Descriptive data summary of key variables in Study 1

	Mean	SD	1	2	3	4	5	6	7	8	9	10	11
1. Age	38.89	5.68											
2. Female	0.27	0.44	−0.12**										
3. Non-Caucasian	0.47	0.50	−0.11*	0.06									
4. Finance industry	0.37	0.48	−0.14**	0.06	0.05								
5. No. of different sex contact	6.68	5.16	−0.05	0.67***	0.04	0.04							
6. No. of different race contact	6.62	6.61	−0.09	0.18***	0.62***	0.06	0.25***						
7. No. of strong ties	15.74	4.86	−0.05	0.07	0.02	0.02	0.21***	0.07					
8. Network size	21.44	4.12	0.01	0.10*	−0.04	−0.04	0.32***	0.17**	0.62***				
9. Network density	0.33	0.18	0.03	0.10*	−0.12*	0.06	0.10*	0.03	−0.12**	−0.09*			
10. Promotion effectiveness	4.08	0.53	−0.07	0.15***	−0.11*	−0.03	0.11*	−0.01	0.13**	0.05	0.07		
11. Prevention effectiveness	3.58	0.84	−0.03	0.12**	0.01	0.00	0.09*	0.01	0.02	0.01	0.00	0.05	
12. Life satisfaction	4.74	1.03	−0.06	0.09*	−0.14***	−0.03	0.07	−0.04	0.10**	0.01	0.15***	0.46***	0.15***

*** *p* < .001; ** *p* < .01; * *p* < .05

#### Results for overall network density

We first tested the main effect of network density, then the main effect of regulatory focus, and then their interaction terms. We always tested the effects of promotion motivation and prevention motivation simultaneously, which allowed us to control for the common variance of these two motivation systems and to assess their unique effects. We also standardized network-density and regulatory-focus variables before calculating the interaction (Aiken and West 1991).

Table 3 (see Appendix) summarizes the OLS regression analyses predicting general life satisfaction. In Model 1, we first tested the main effects of overall network density, controlling for the demographic variables, the number of strong ties, and overall network size. Consistent with past research, the number of strong ties had a significant and positive main effect on life satisfaction (*b* = .04, *p* < .001), as well as network density (*b* = .14, *p* < .002).^2^ Next, Model 2 showed a significant main effect of regulatory foci (*b*_promotion_ = .44, *p* < .001; *b*_prevention_ = .13, *p* < .001) on life satisfaction. While both effects were significant, we replicated the past finding (e.g., Ferris et al. 2013) that promotion effectiveness showed a stronger significant effect size on life satisfaction than prevention effectiveness (Δb = 0.31, *p* < .05, Cumming 2009).^3^ Model 3 tested the central predictions of our study—the interaction effect between overall network density and the two regulatory foci.

**Fig. 1:**
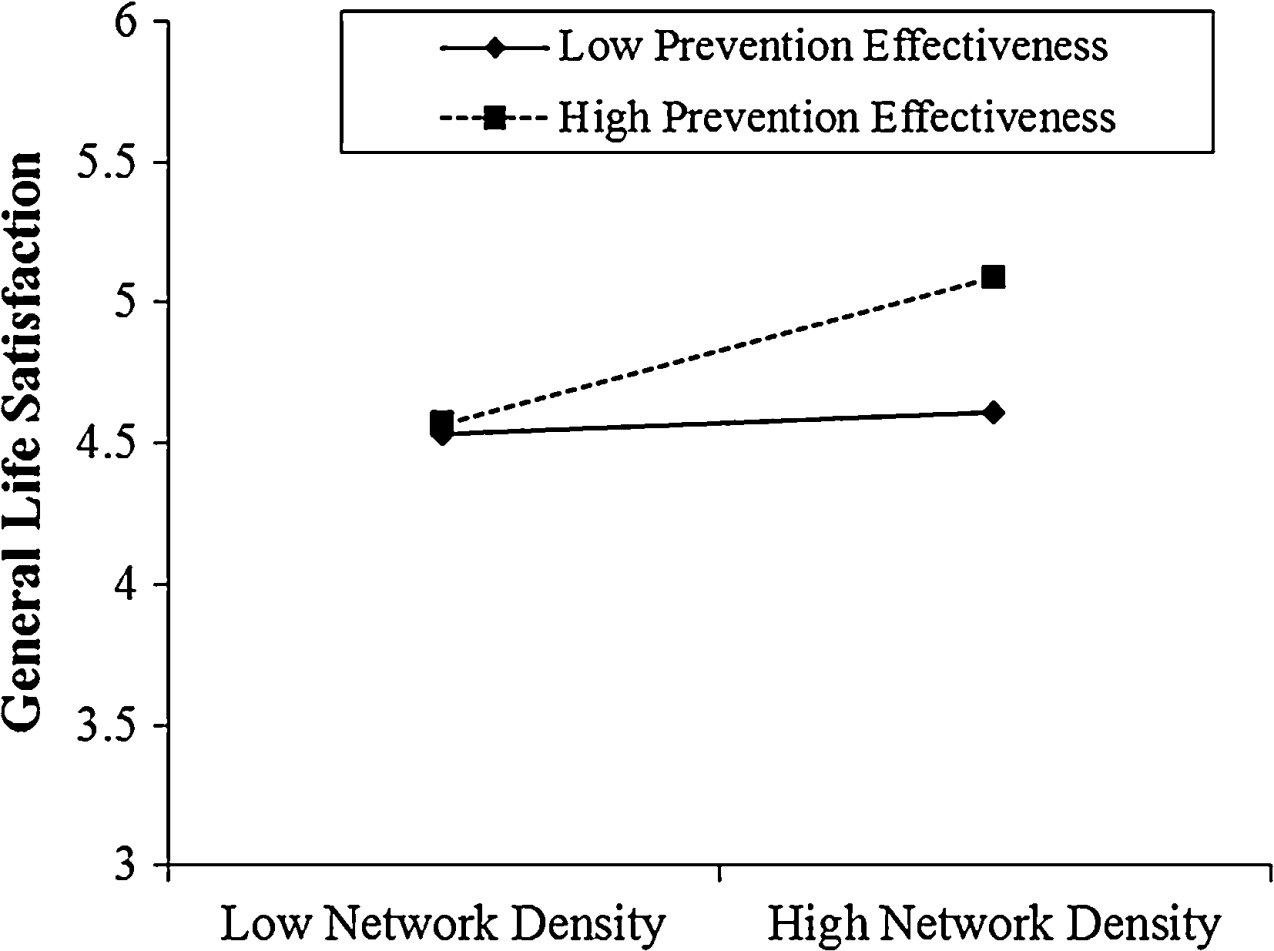
Interaction effect between prevention effectiveness and overall network density in predicting general life satisfaction (Study 1)

Consistent with our hypothesis, we observed a significant interaction effect between network density and prevention effectiveness (*b* = .11, *p* < .024). Figure 1 plots values representing plus and minus one standard deviation from the means on prevention effectiveness and network density to demonstrate the interaction effect. For managers with prevention effectiveness one standard deviation above the mean, network density had a strong positive effect on life satisfaction (*b* = .16, *p* < .009). For managers with prevention effectiveness one standard deviation below the mean, network density effect was non-significant (*p* = .45). The interaction effect between network density and promotion effectiveness was not significant (*p* = .87).

#### Additional analysis

Given the non-significant finding for the interaction between promotion effectiveness and network density, we wondered if it mattered that the density in our respondents’ professional networks depended partly on structural influences from their jobs. As participants in our study were from different organizations and since we did not have detailed job descriptions for each participant and thus could not directly control for these organization-specific variances, we investigated our hypotheses by separating the overall network density into three components: density inside the organization, density between insiders and outsiders, and density solely among alters outside the organization. If our conjecture is correct, we are more likely to observe interaction effects between regulatory effectiveness and network density outside the organization.

When we collected the network data, we asked participants to indicate whether each alter was within or outside of his or her organization. Based on this information, we used the same network density formula reported above to calculate three network density scores: network density within the organization (where i and j were inside the same organization as the participant), network density outside the organization (where i and j were outside the organization of the participant), and network density across the organization boundary (where i was inside the organization and j outside, or vice versa). Overall network density had positive associations with network density across the organization boundary (r = 0.43, *p* < .001) and network density outside the organization (r = .67, *p* < .001), as well as with network density within the organization boundary (r = .35, *p* < .011).

**Fig. 2:**
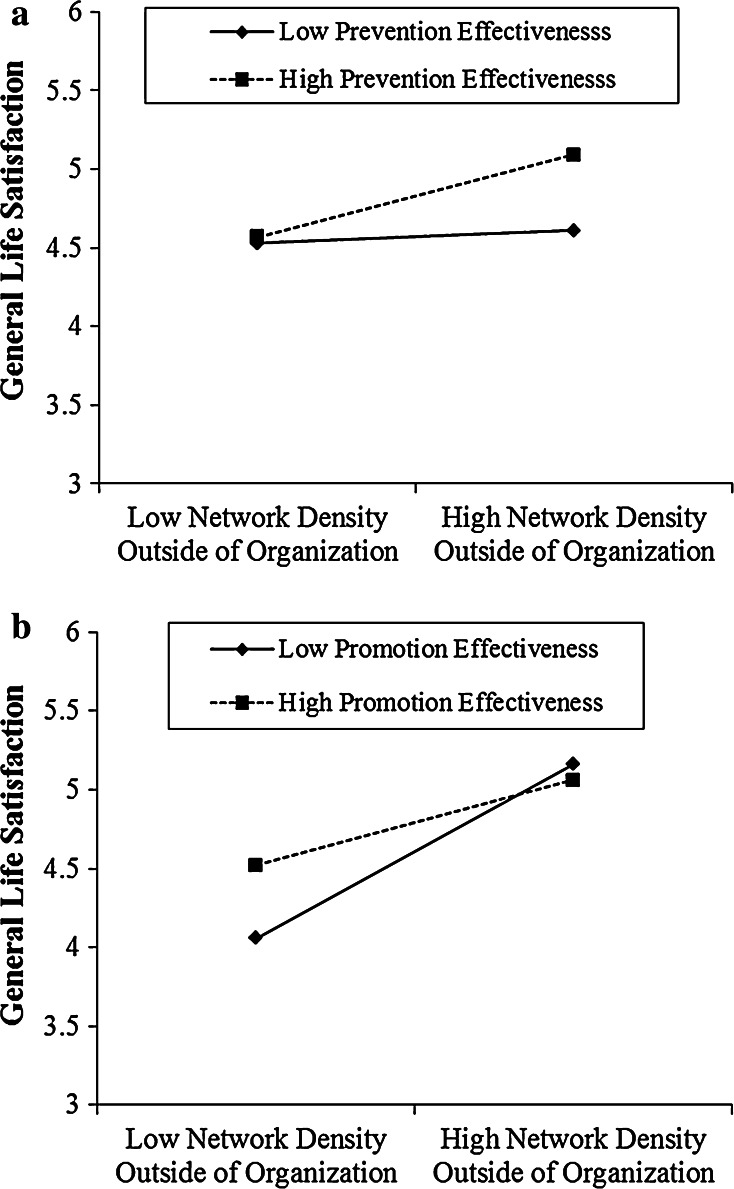
**a** Interaction effect between prevention effectiveness and network density outside the organization in predicting general life satisfaction (Study 1). **b** Interaction effect between promotion effectiveness and network density outside the organization in predicting general life satisfaction (Study 1)

Next, in Model 4, we examined the effect of network density outside the organization. We observed a significant main effect of network density (*b* = .09, *p* < .044), as well as significant interaction effects with both prevention effectiveness (*b* = .09, *p* < .05) and promotion effectiveness (*b* = −.14, *p* < .003). The prevention effectiveness interaction with density outside the network, illustrated in Fig. 2a, represents a replication of the pattern of the interaction between overall network density and prevention effectiveness.

More importantly, now we observed strong evidence for an interaction effect between promotion effectiveness and network density outside the organization (*b* = −.14, *p* < .003). As illustrated in Fig. 2b, network density outside the organization had no significant effect among managers who were one standard deviation above the average promotion effectiveness score, but had a significant positive effect among managers who were one standard deviation below the average promotion effectiveness score (*b* = .22, *p* < .001). That is, participants with high promotion effectiveness reported a high level of life satisfaction regardless of the network density levels, but those with low promotion effectiveness suffered significantly in a low density network.

In Model 5, we tested the effects of density inside the organization. It showed a significant main effect (*b* = .09, *p* < .037) on life satisfaction, but no interaction effect with either promotion effectiveness or prevention effectiveness. Model 6 showed the effect of network density across the organizational boundary, displaying a marginally significant main effect of cross-boundary density (*b* = .08, *p* = .055) and a significant interaction effect with prevention effectiveness (*b* = .10, *p* < .028). This prevention interaction pattern is similar to the results based on overall density score.

Overall, evidence from Study 1 shows that network density can significantly impact life satisfaction, moderated by two distinct types of self-regulatory effectiveness. Consistent with our hypothesis, prevention effectiveness moderates the effect of overall network density (including both network density scores outside of the work organization and across the organization boundary) on life satisfaction.

However, results on the interaction effect between promotion effectiveness and network density outside of the organization were not what we predicted. Originally, we predicted that participants with high promotion effectiveness would benefit more from a low density than a high density network. However, our results suggest that participants with high promotion effectiveness report a higher level of life satisfaction irrespective of their network density levels. This finding could derive from the fact that promotion-focused people have an optimism bias (Grant and Higgins 2003)—they see what they want to see. People with high promotion effectiveness are likely to hold a rose-colored view across various situations, and thus it is possible that they perceive valuable resources from both the low and high density networks.

The promotion-related interaction with network density that we found instead was the converse of our hypothesis: participants with low promotion effectiveness suffered significantly in low density networks. A possible explanation for this is that because a low density network signals opportunities for achievement and gains, such promotion-serving opportunities highlight the promotion failure experienced by participants with low promotion effectiveness; that is, they are promotion ineffective despite being given support in the service of promotion effectiveness. If so, this would still be consistent with that part of our theoretical argument that a low density network provides promotion-serving supports (Hypothesis 2b). But such support, instead of enhancing life satisfaction for promotion effective individuals, brings a sense of failure to promotion ineffective individuals.

Drawing on the findings from Study 1 and the above interpretations of them, in Study 2 we directly measured the degree to which participants perceive promotion-serving supports and prevention-serving supports from their social networks. Our aims were to test whether individuals with high prevention effectiveness perceive more prevention-serving support in high density networks (beneficial to them in high density networks); whether individuals with low promotion effectiveness perceive more promotion-serving support in low density networks (detrimental to them in low density networks); and whether individuals with high promotion effectiveness perceive valuable promotion-serving resources from both the low and high density networks (beneficial to them across networks). In this way, we can investigate whether these perceptions of supports explain the links between network density and life satisfaction identified and interpreted in Study 1.

## Study 2

Study 2 extends the findings in Study 1 in two important ways. First, we examined whether high-density networks are indeed more likely to provide support that addresses issues particularly relevant to prevention self-regulation, whereas low-density networks are more likely to provide support that addresses issues particularly relevant to promotion self-regulation. Thus, we measured the extent to which participants obtain prevention-serving versus promotion-serving support from their social networks. In light of the findings from Study 1, we further predicted that the prevention-serving support has a positive association with life satisfaction among high prevention effective individuals. However, the promotion-serving support would have a negative association with life satisfaction among individuals with low promotion effectiveness. We conducted moderated mediation analyses to test whether prevention-serving support mediates the high-density network effect among prevention effective individuals and whether promotion-serving support mediates the low-density network effect among promotion ineffective individuals.

Second, we further extend Study 1 by including a comprehensive list of individual difference variables that have previously been demonstrated to be important in the social network literature. First, we included self-monitoring, which considers individual differences between those who are attuned and responsive to the situated expectations of others versus those who insist on being themselves despite current social expectations (Snyder 1974; Snyder and Gangestad 1986). Past research has argued that network variables should display stronger predictive power among high self-monitors in predicting instrumental outcomes, such as job performance (e.g., Mehra et al. 2001). However, we do not expect self-monitoring to moderate the effect of network density on well-being outcomes.

Another individual difference variable included in this study is need for closure. Some evidence has shown that need for closure affects people’s perception of network structure (Flynn et al. 2010). In that study, individuals with a high need for closure were more likely to assume that their social contacts were connected to each other. The inclusion of the need for closure measure addressed the possibility that our network density result was not driven by actual network density differences but by perceived connectedness stemming from individual levels of need for closure.

Finally, another concern is that our findings relating regulatory focus to life satisfaction through network density might be due to associations between chronic regulatory focus and other general personality traits. For example, past research has shown that big-five personality variables are strongly associated with life satisfaction (e.g., DeNeve and Cooper 1998; Diener and Lucas 1999; Schimmack et al. 2004). On the other hand, regulatory focus has critically mediates the relationship between personality traits and various satisfaction indexes (see Lanaj et al. 2012). Controlling for the big-five personality thus can further clarify whether the observed effect of self-regulation effectiveness on life satisfaction are unique to regulatory focus and not redundant with personality. Thus, we controlled for the Big-Five personality factors: extraversion, emotional stability, openness to change, agreeableness, and conscientiousness (Goldberg 1992).

### Participants and design

Two hundred and fifty-two participants were recruited from a behavioral research lab located in central London, UK (58 % female; *M*_age_ = 25. 63, *SD* = 7.54). Of these, 38.9 % were White British, 26.4 % were Asian British (mainly Chinese and Indian), 12.4 % were African British, and the rest were of other races (mostly Europeans, Middle Eastern). 78 % of the participants were students from universities in central London, and the rest held a full-time job in the local area (mainly staff from the university).

The study consisted of two parts. First, participants completed an electronic survey about their social networks. They were asked to list up to 24 contacts who were important in their social networks and then provide the relevant information following the same format as in Study 1. Second, participants completed a three-section online survey. Section one of the online survey consisted of a list, in random order, of individual difference measures, including regulatory focus orientation, self-monitoring, need for closure, and the Big-Five Inventory. In section two, participants rated the degree to which they obtained both promotion-serving and prevention-serving support from their social networks. Finally, participants rated their general life satisfaction (Diener et al. 1985) on the same three items used in Study 1, as well as provided the demographic information.

### Promotion-serving versus prevention-serving support

To capture the distinct support functions of high- and low-density networks, we created two three-item measures, to which participants responded on a 6 point Likert scale from 1(*strongly disagree)* to 6 (*strongly agree*). The prevention-serving support measure included the following items: “I often fear that I cannot control my reputation in my social networks (reverse coded).” “I trust my social network contacts.” and “My social networks effectively facilitate me in fulfilling my responsibilities and obligations.” (α = 0.77). The promotion-serving support measure included the following items: “My social network contacts constantly inspire me to reach my ideal self.” “My social network constrains my future development (reverse coded).” and “My social network inspires me to become more creative.” (α = 0.62). Exploratory factory analysis showed that these six items loaded onto two separated factors. Eigenvalue of promotion-serving support is 1.33, and eigenvalue of prevention-serving support is 1.63. These two factors were not correlated (r = .04, *p* > .5).

### Individual difference measures

#### Big five personality

In measuring the Big-Five Inventory, we used Goldberg’s (1992) terminology. Following the stem “I see myself as …”, participants responded on a 5-point scale ranging from 1(*strongly disagree)* to 5 (*strongly agree*). The scale included 48 items on the following traits: conscientiousness (*M* = 3.69, *SD* = 0.53, α = 0.67, 8 items), agreeability (*M* = 3.38, *SD* = 0.57, α = 0.78, 9 items), emotionally stability (*M* = 3.28, *SD* = 0.81, α = 0.68, 7 items), extraversion (*M* = 3.20, *SD* = 0.78, α = 0.81, 6 items), and openness to new experiences (*M* = 3.79, *SD* = 0.62, α = 0.69, 6 items).

#### Need for closure

Participants completed the Need for Closure (NFC) Scale developed and validated by Webster and Kruglanski (1994). Items were rated using a 6-point scale ranging from 1 (*Strongly disagree*) to 6 (*Strongly agree*). The NFC Scale consists of 42 items. Two sample items are “I don’t like situations that are uncertain” and “I think it is fun to change my plans at the last moment” (reverse coded). Embedded in the scale are five items assessing social desirability. Following the guidelines outlined by Webster and Kruglanski, we summed a “lie score” for these five items and removed individuals from the sample who received a score of 15 or higher (*N* = 14).

#### Self-monitoring

We assessed the participants’ self-monitoring tendencies with the Self-Monitoring Scale (SMS; Snyder 1974). It consists of 25 self-descriptive statements intended to capture several elements of social adroitness, including concern with situational appropriateness, attention to social cues, and ability to control expressive behavior. Each of the items (e.g., “I’m not always the person I appear to be.”) was rated using true or false responses. We summed the responses to create an overall score for self-monitoring (*M* = 13.17, *SD* = 3.61).

#### Control variables

We controlled for participants’ age, sex, and employment status (1 = fulltime employed, 0 = otherwise). ANOVA revealed that African British participants reported a significantly lower level of life satisfaction than White British, Asian British, and participants of other ethnic categories, *F*(1, 252) = 4.88, *p* < .003. Therefore, in the main analysis we included African British as an ethnicity control (1 = yes, 0 = no).^4^

### Results

#### Predicting life satisfaction

Given that more than three-quarters of our participants in Study 2 were fulltime students, the concern of job features as a potential confounding factor, as it was in Study 1, was no longer an issue. Thus, breaking network density into within- between- and outside- organization categories was not necessary for participants who were not embedded in a work organization structure. We tested our hypotheses by using only the overall network density score. Table 2 summarizes the descriptive statistics and zero-order correlation among the variables. Table 4 (see Appendix) summarizes the regression results.

**Table 2:** Descriptive data summary of key variables in Study 2

	Mean	SD	1	2	3	4	5	6	7	8	9	10
1. Female	1.58	0.49										
2. Age	25.63	7.54	−0.16*									
3. British African	0.12	0.33	−0.17**	0.18**								
4. Fulltime employed	1.78	0.42	−0.03	−0.38***	−0.02							
5. Network size	15.96	5.66	−0.01	−0.05	−0.09	0.11						
6. Network density	0.40	0.25	0.04	−0.05	0.03	−0.09	−0.12*					
7. Promotion effectiveness	3.59	0.63	0.02	−0.01	−0.08	0.05	0.15*	0.04				
8. Prevention effectiveness	3.19	0.82	0.08	0.03	0.00	0.14*	0.04	−0.04	0.06			
9. Promotion-serving support	3.97	0.65	0.03	−0.13*	−0.10	0.17**	0.08	0.09	0.18**	0.00		
10. Prevention-serving support	5.06	0.93	−0.05	0.15*	0.13*	−0.03	0.03	−0.19**	0.20***	0.05	0.04	
11. Life satisfaction	4.36	1.33	0.05	−0.28***	−0.19**	0.10	0.14*	0.12*	0.45***	0.14*	0.20**	0.05

*** *p* < .001; ** *p* < .01; * *p* < .05

First, we regressed network density on life satisfaction, controlling for each participant’s sex, age, ethnicity, employment status, and network size (Table 4 in Appendix, Model 1). Consistent with Study 1, there was a significant positive effect of higher network density (*b* = .18, *p* < .04). Among the control variables, younger participants (*b* = −.05, *p* < .001) and African British participants (*b* = −.47, *p* < .03) reported lower life satisfaction. Next, we added regulatory focus variables in Model 2. Again, consistent with Study 1, both higher promotion effectiveness (*b* = .55, *p* < .001) and higher prevention effectiveness (*b* = .17, *p* < .02) showed significant main effects on life satisfaction. Again, we replicated findings from Study 1 and past research (Ferris et al. 2013) that promotion effectiveness showed a stronger effect size (Δb = 0.38, *p* < .05, Cumming 2009).^5^. In Model 3, we tested the interaction terms between network density and the two regulatory focus measures. Consistent with our hypothesis, we found a positive interaction effect between network density and prevention effectiveness (*b* = .18, *p* < .045), and a negative interaction effect between network density and promotion effectiveness (*b* = −.19, *p* < .022).

**Fig. 3:**
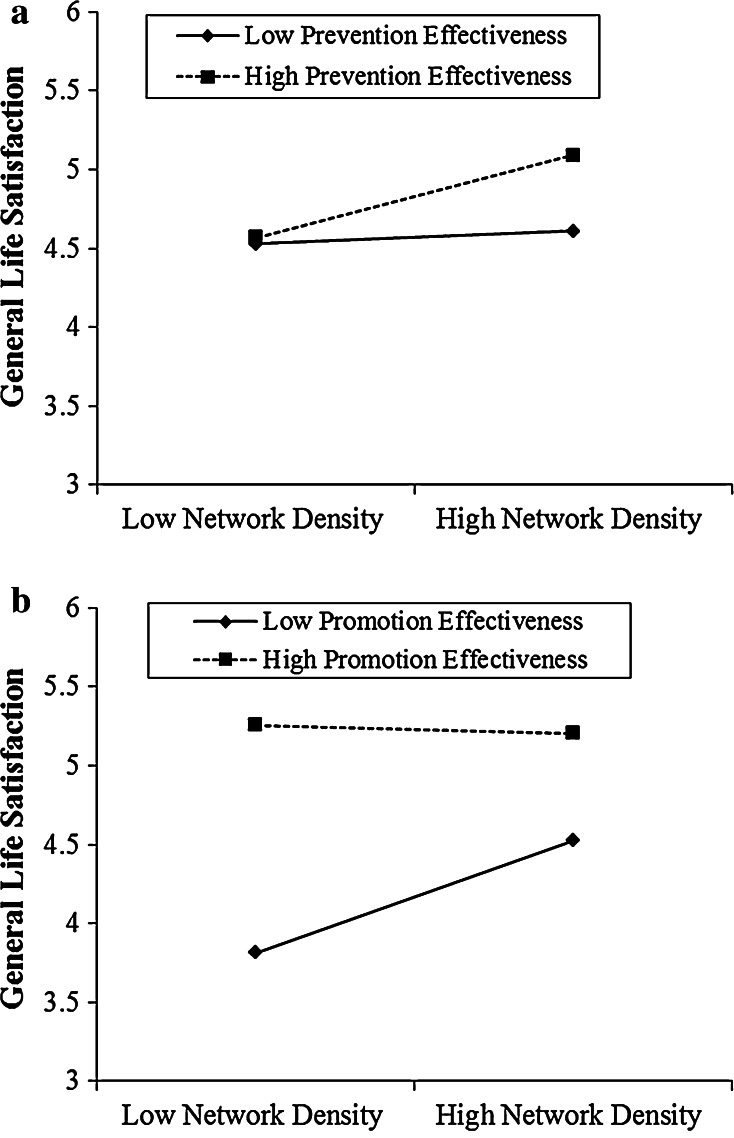
**a** The interaction effect between network density and prevention effectiveness on life satisfaction (Study 2). **b** The interaction effect between network density and promotion effectiveness on life satisfaction (Study 2)

Figure 3a depicts the interaction effect between prevention effectiveness and network density. It plots the simple slope of network density at two values of prevention effectiveness. Network density showed no effects on life satisfaction among participants with low prevention effectiveness (−1 SD: *p* = .90), but a significant positive effect among participants with high prevention effectiveness (+1 SD: *b* = .33, *p* < .001). In other words, we found a facilitating effect of high network density for high prevention effective individuals. When embedded in a high (vs. low) density network, high prevention effective individuals showed significantly higher life satisfaction than low prevention effective individuals.

Figure 3b depicts the interaction effect between promotion effectiveness and network density, showing a very different pattern. A test of simple slopes across the two levels of promotion effectiveness revealed a null effect between network density and life satisfaction among participants with high promotion effectiveness (+1 SD: *p* = .9); but a significant effect of network density on life satisfaction for promotion ineffective participants (−1 SD: *b* = .34, *p* < .004). Whereas individuals with high promotion effectiveness seem highly satisfied within both low and high density networks, those with low promotion effectiveness suffer in a low density network. Replicating the pattern of interaction found in Study 1, participants with low promotion effectiveness reported a significant lower level of life satisfaction (*M* = 3.82) than participants with high promotion effectiveness (*M* = 5.26) under low network density (−1 SD), *p* < .001.

#### Predicting promotion-serving and prevention-serving support

**Fig. 4:**
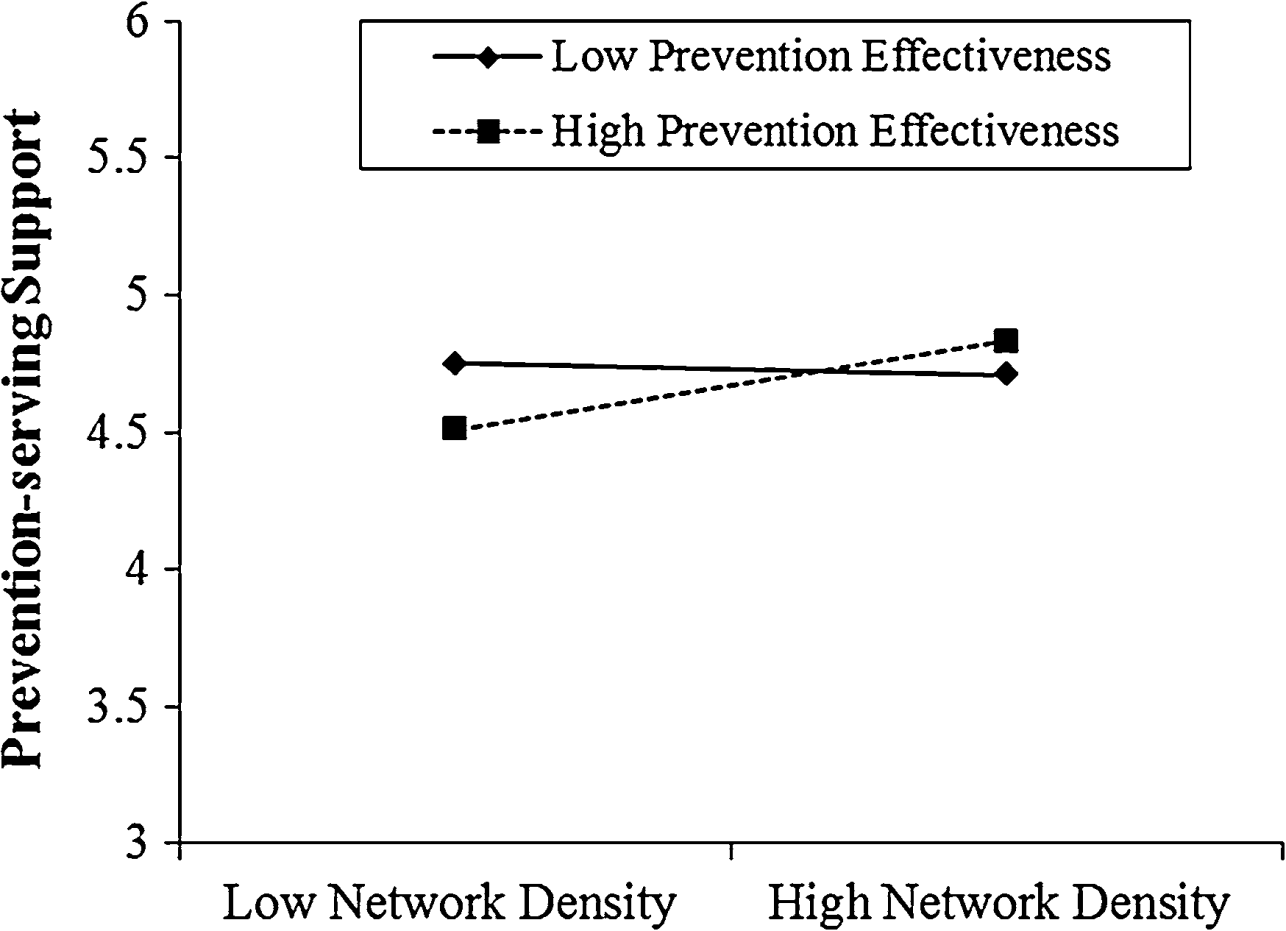
The interaction effect between prevention effectiveness and network density on prevention-serving support function (Study 2)

Next, we tested the effect of network density and regulatory focus on the support functions of social networks. We first regressed network density and prevention effectiveness on perceived prevention-serving support, including the control variables and promotion effectiveness (Table 4 in Appendix, Model 4). Neither prevention effectiveness nor network density had a significant main effect. However, the interaction effect between these two was significant (Table 4 in Appendix, Model 5, *b* = .09, *p* < .043). Network density showed no effect on perceived prevention-serving support among participants low in prevention effectiveness (−1 SD: *p* = .87), but a significant positive effect among participants high in prevention effectiveness (+1 SD: *b* = .14, *p* < .018). Consistent with our hypothesis, individuals with high prevention effectiveness are more likely to report receiving prevention-serving support in a high (vs. low) density network, see Fig. 4. In addition, there was also a significant main effect of promotion effectiveness (b = .09, *p* < .014), supporting the earlier conjecture that individuals with high promotion effectiveness would show an optimism bias—see the world with rose-colored glasses—and thus generally report a higher level of perceived support.

We repeated the same steps to test the effects of network density and promotion effectiveness on perceived promotion-serving support, including the control variables and prevention effectiveness (Table 4 in Appendix, Model 6). We found two distinct main effects. Participants with lower network density reported that their social networks provided significantly more promotion-serving support (*b* = −.19, *p* < .002). In addition, and independent of network density, participants with high (vs. low) promotion effectiveness reported that their social networks provided significantly more promotion-serving support (*b* = .21, *p* < .001), consistent with the notion that high promotion effective individuals see the world as they want it to be.

Next, we tested the interaction effect between network density and promotion effectiveness. The effect of this interaction on promotion-serving support was only marginally significant (Table 4 in Appendix, Model 7, *b* = .13, *p* = .084). This finding has two significant implications. First, consistent with the notion of optimism bias, individuals with high promotion effectiveness perceive a high level of promotion-serving support regardless of the density of their networks. Second, it provides the first piece of direct evidence that individuals with low promotion effectiveness also perceive a high level of promotion-serving support from a low density network, given the main effect of network density that was found. Thus, perceived promotion-serving is a potential mediator to explain the link between low promotion effectiveness and low life satisfaction under low density networks. That is, the promotion-serving supports found in a low density network (e.g., “inspiring me to reach my ideal self”, “be creative”) might ironically reduce the life satisfaction among low promotion effective individuals (“I fail in promotion effectiveness even when I am receiving social support to be promotion effective”).

#### The effect of regulatory focus support functions on life satisfaction

Next, we tested whether the two support functions mediate the interaction effect between network density and regulatory focus effectiveness on life satisfaction. We first established the effect of the support functions on life satisfaction. Model 8 showed a significant main effect of prevention-serving support (*b* = .31, *p* < .017), but a non-significant main effect of promotion-serving support (*b* = .15, *p* = .102). Model 9 further showed that there was a significant interaction effect between prevention effectiveness and prevention-serving support (*b* = .16, *p* < .034), as well as a significant interaction effect between promotion effectiveness and promotion-serving support (*b* = .23, *p* < .02). These two interaction terms are the critical mediators in the subsequent analyses.

**Fig. 5:**
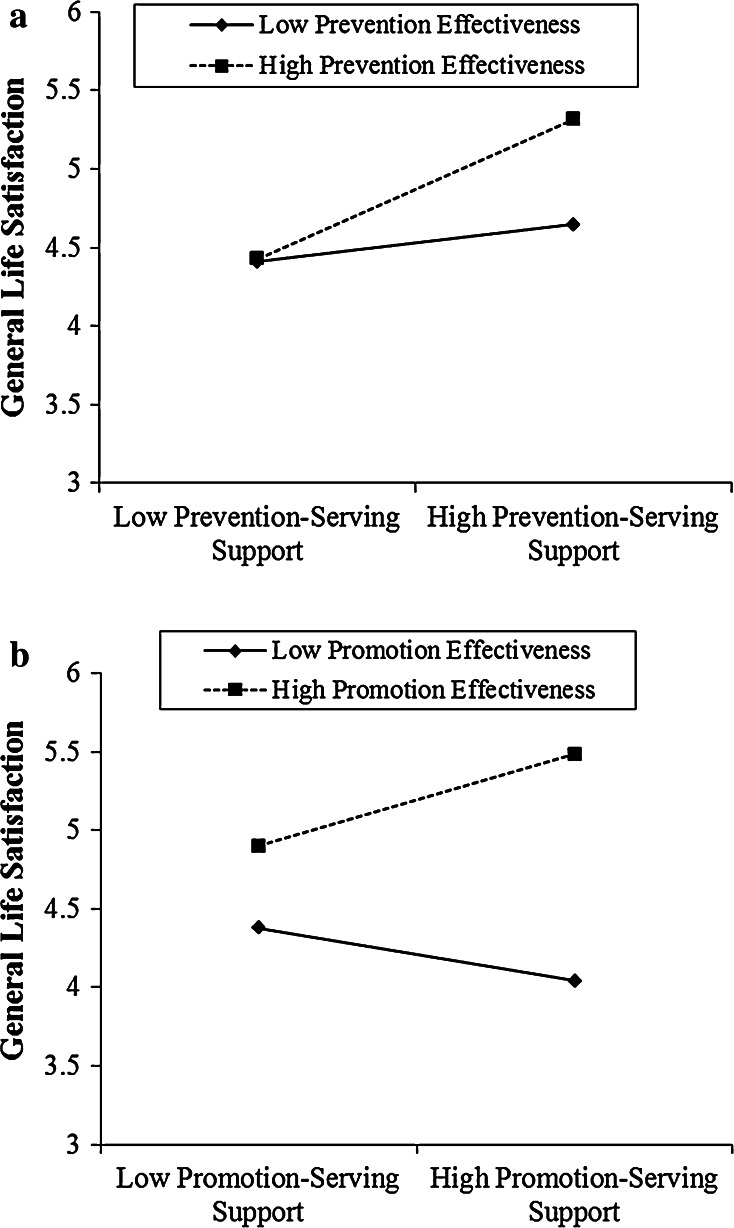
**a** The interaction effect between prevention-serving support function and prevention effectiveness on life satisfaction (Study 2). **b** The interaction effect between promotion-serving support function and promotion effectiveness on life satisfaction (Study 2)

We conducted further analyses to unpack the nature of the interaction terms. First, prevention-serving support had a significant positive effect on life satisfaction among participants with high prevention effectiveness (+1 SD: *b* = .28, *p* < .012), which is what would be expected from a regulatory fit (Higgins 2000). There was no such effect for participants with low prevention effectiveness (−1 SD: *p* = .63) (see Fig. 5a). On the other hand, Fig. 5b shows that promotion-serving support had a significant positive effect on life satisfaction among participants with high promotion effectiveness (+1 SD: *b* = .27, *p* < .024), which is again what would be expected from a regulatory fit (Higgins 2000). In addition, as also shown in Fig. 5b, promotion-serving support had a marginally significant negative effect on life satisfaction among participants with low promotion effectiveness (−1 SD: *b* = −.19, *p* = .07). This suggests that promotion-serving support is like a double-edged sword: It is beneficial to individuals with high promotion effectiveness, but detrimental to those with low promotion effectiveness.

#### Mediation analysis

**Fig. 6:**
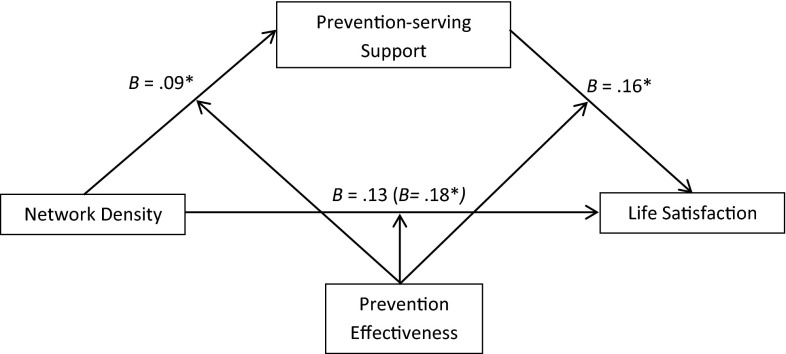
Mediation analysis demonstrating that the effect of network density on life satisfaction was mediated by prevention-serving support among high prevention effective participants. *Note* ***p < .001; **p < .01; *p < 0.05

We first tested whether prevention-serving support mediates the effect of network density on life satisfaction among participants with high prevention effectiveness. We used the bootstrapping method (with 1000 iterations) provided by Preacher et al. (2007) to test this moderated mediation hypothesis. In this analysis, prevention-serving support served as the mediator, prevention effectiveness served as the moderator, and network density served as the independent variable. We controlled for the same list of control variables, promotion-serving support, and promotion effectiveness. We used Model 59 under the PROCESS macro for the mediation analysis, in which prevention effectiveness moderated all three links in the model (i.e., the network density to prevention-serving support effect, the network density to life satisfaction effect, and the prevention-serving support to life satisfaction effect). Figure 6 summarizes this mediation model.

The interaction effect between network density and prevention effectiveness becomes only marginally significant (*b* = .13, *p* = .069) after controlling for the mediator (the prevention-serving support X prevention effectiveness interaction), while the effect of the mediator remains significant (*b* = .17, *p* < .019). The 95 % corrected confidence intervals for the size of the indirect effect of network density excluded zero (95 % CI [0.0166, 0.1307]) among participants with high prevention effectiveness, but not among those with low prevention effectiveness (95 % CI [−0.0174, 0.0243]). This analysis revealed a significant moderated mediation effect. Consistent with our hypothesis, individuals with high prevention effectiveness showed a significantly higher level of life satisfaction in high-density networks that are perceived to provide stronger prevention-serving support.

**Fig. 7:**
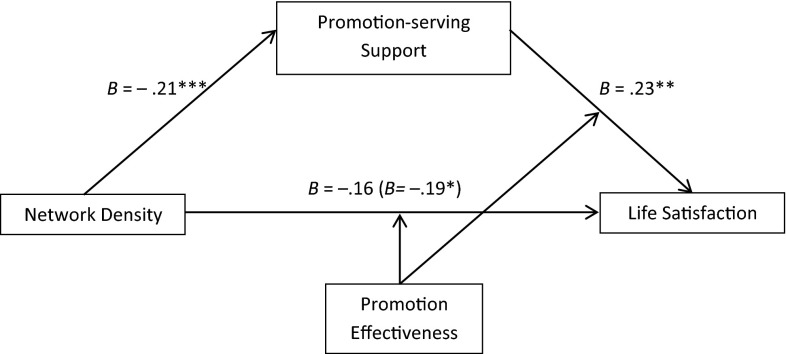
Mediation analysis demonstrating that the effect of network density on life satisfaction was mediated by promotion support among high promotion effective participants. *Note* ***p < .001; **p < .01; *p < .05

Next, we tested the mediation effect of promotion-serving support. Specifically, we hypothesized that promotion-serving support mediates the effect of network density on life satisfaction among high promotion effective participants. We used the same method as above: promotion-serving support function served as the mediator, promotion effectiveness served as the moderator, and network density served as the independent variable. We controlled for the same list of control variables, prevention-serving support, and prevention effectiveness. Because there was only a main effect of network density on promotion-serving support, we only treated promotion effectiveness as a moderator on the link between promotion-serving support and life satisfaction, and the link between network density and life satisfaction, using Model 15 under the PROCESS macro for the mediation analysis. Figure 7 summarizes the mediation model.

The interaction effect between network density and promotion effectiveness becomes only marginally significant (*b* = −.16, *p* = .068) after controlling for the mediator (promotion-serving support X promotion effectiveness interaction), while the effect of the mediator on life satisfaction remained significant (*b* = .20, *p* < .02). The 95 % corrected confidence intervals for the size of the indirect effect of network density excluded zero (95 % CI [−0.1312, −0.0028]) among participants with low promotion effectiveness, but not among those with high promotion effectiveness (95 % CI [−0.0190, 0.0819]). This analysis revealed a significant moderated mediation effect. Consistent with our interpretation of the results of Study 1, individuals with low promotion effectiveness showed a significantly lower level of life satisfaction in low-density networks that are perceived to provide promotion-serving support. That is, despite being in a low density network that is providing support to be promotion effective, these individuals are still promotion ineffective. It is not surprising that this would reduce life satisfaction.

It should also be noted that the hypothesized benefit of the low (vs. high) density network for individual with high promotion effectiveness did not occur because these individuals perceived they were receiving the promotion support they wanted in the high density network as well. Importantly, this does not mean there was no regulatory fit effect for them. As reported above, there was a significant interaction effect between perceived promotion-serving support and promotion effectiveness: promotion serving support had a significantly stronger effect on life satisfaction among individuals with high promotion effectiveness.

#### Other individual difference measures

We repeated the same series of analysis above by controlling for the additional individual difference variables respectively, including the Big-Five personality variables, self-monitoring, and need for closure. The pattern of results reported above remained the same. We did not identify any significant interaction effects from need for closure or from self-monitoring.

We did, however, observe a significant main effect of emotional stability (*b* = .36, *p* < .001) and conscientiousness (*b* = .31, *p* < .001) on life satisfaction, as well as a significant interaction effect between extraversion and network density (*b* = −.27, *p* < .009).^6^ It is worth noting that a large body of research has examined the relationship of the Big-Five personality factors and subjective well-being (e.g., Costa and McCrae 1980; Headey and Wearing 1992; Tellegen 1985; Watson and Clark 1992; see Lucas and Fujita 2000, for a meta-analytical review). Personality variables are usually stronger predictors to affective-based well-being measures than cognitive-based well-being measures, such as the Satisfaction with Life Scale (Steel et al. 2008). Because the pattern of network density and regulatory foci interactions remained the same after controlling for the significant personality effects, we are confident that effects of regulatory focus were not confounded by associated personality factors.

Overall, Study 2 provides further evidence for the interaction effect of regulatory effectiveness and network density on life satisfaction. More importantly, we demonstrated that the effects of high- and low-density networks have distinct implications for two different types of self-regulatory effectiveness. High-density networks are better at providing trust and ensuring the fulfillment of obligations, which are essential for the well-being of individuals with high prevention effectiveness. By contrast, low-density networks are better at providing opportunities for creative inspiration and personal development, which actually reduces well-being among individuals with low promotion effectiveness.

## General discussion

Drawing on the research of social capital and self-regulation, we proposed that a new form of fit –regulatory focus orientation and network density—have significant implications for individuals’ well-being. Three sets of results emerged from our analyses. First, there was a strong fit between high prevention effectiveness and high density network. High-density networks had a significant positive effect on life satisfaction among high prevention effective individuals. By contrast, there was a strong non-fit between individuals with low promotion effectiveness and low density network. Low-density networks had a significant but negative effect on life satisfaction among low promotion effective individuals. Across the two samples, there was generally a positive main effect of network density. In part, the main effect of network density could be unpacked as the positive effect of high density network on high prevention effective individuals and the negative effect of the low density network on low promotion effective individuals. In our study, we did not observe any non-fit effect from the high density network nor any fit effect from the low density network.

Critically, our findings also highlight the importance of distinguishing two forms of perceived social support and their fit with either a promotion or a prevention motivation system. Specifically, high prevention effective individuals were more likely to perceive prevention-serving support from low density network, and the perceived prevention-serving support in turn had a stronger positive effect on life satisfaction among individuals with high prevention effectiveness. On the other hand, both high and low promotion effective individuals were more likely to perceive promotion-serving support from a low density network than from a high density network. However, the perceived promotion-serving support has a positive effect on life satisfaction among individuals with high promotion effectiveness but a negative effect among individuals with low promotion effectiveness. The perceived promotion-serving support is detrimental to individuals with low promotion effectiveness, presumably because they recognize that they are promotion ineffective despite receiving strong promotion support in their low density networks.

Last but not least, we replicated the past results that there was a strong main effect of promotion effectiveness on life satisfaction (Grant and Higgins 2003). In particular, the effect size of promotion effectiveness on life satisfaction was significantly stronger than the effect size of prevention effectiveness. In addition, individuals with high promotion effectiveness showed a significant higher level of life satisfaction than individuals with low promotion effectiveness in both low and high density networks. That is, individuals with high promotion effectiveness do not show differentiated fits with different levels of network density but a general positive bias on life satisfaction. This is because their “rose-colored glasses” makes them perceive receiving promotion-serving support in both low *and* high density networks—their optimism bias of seeing what they want to see (Grant and Higgins 2003).

Overall, our theoretical framework and the empirical findings have important implications for research on well-being, social networks, and self-regulation. First, our results offer a promising direction for studying the psychology of social networks by focusing on individual well-being outcomes. Research on the consequences of social networks has largely focused on performance and instrumental outcomes (Borgatti and Cross 2003; Brass et al. 2004; Kilduff and Brass 2010). Few studies have asked whether network structure can affect individuals’ well-being. Yet, well-being is a major concern of people across the world, and presumably, the end to which “instrumental” outcomes like income, promotions and job performance, act as means (Diener et al. 1999). Traditionally, social networks have often been treated as pipes for various resources/opportunities (Podolny 2001). In our view, resources or opportunities offered by different network structures may be differentially valued by individuals with distinct regulatory-focus orientations. By specifying the interplay between network density and individuals’ self-regulatory systems, our model provides a psychological framework through which to analyze the effect of social networks on individual well-being.

Second, the interplay between individuals’ regulatory focus and social network structure provides new insights for understanding the dynamic nature of social networks and the role of individual agency in understanding the consequence of social networks. In this regard, prior research has shown that people differ significantly in their help-seeking preferences. For example, people at different status levels spontaneously activate different subsections of their networks when faced with job threat (Smith et al. 2012). People with low status tend to activate smaller and tighter subsections of their networks, whereas people with high status activate larger and less constrained subsections of their networks. Given our findings in the current paper, future studies should examine how individuals with different motivational systems activate different parts of their networks for seeking help. Individuals with high prevention effectiveness, for example, might rely more on the densest parts of their network, whereas individuals with low promotion effectiveness might actually avoid the densest parts of their network.

Third, our findings also contribute to the psychological research on well-being. Thus far, various congruence models have been proposed to study how the person-environment fit can affect individual well-being (Diener and Lucas 1999; Oishi et al. 2007). Yet, a recurring challenge is to capture the contextual factors objectively and effectively. Social network instruments provide a perspective for understanding the effect of social context on well-being. For example, some research has shown that extraverts are happier than introverts when they are with others rather than alone (Costa and McCrae 1980), but other research suggests that extraverts are happier regardless of whether they live alone or live with others, whether they work in social or nonsocial occupations, and whether they are in social situations or alone (Diener et al. 1984). Still other research shows that both extraverts and introverts are happier being in social situations than being alone (Pavot et al. 1990).

In these studies, measures of the sociality level of a context involved asking participants to evaluate the social context through subjective ratings, which may introduce substantial measurement errors. More importantly, calling a context “social” ignores the fact that a social context can involve distinct social network structures. A person, as an ego in a social network, can interact with an alter connected to all other alters in the ego’s network or with an alter uniquely connected with the ego. These interactions may all be counted as social, but they can be qualitatively different. In this regard, social network instruments may provide critical insights for unpacking the psychological features of various social contexts and better understanding which kind of “social” context better fits which kind of person.

### Limitations and future research

The limitations of our studies also need to be highlighted. First, both studies used only egocentric network measures. The strength of the egocentric network is that it is not constrained by a network boundary imposed by researchers, so this study capitalized on the broader scope of egocentric networks and examined network structures both within and across an organization’s boundaries. At the same time, the fact that our participants in Study 1 came from more than 100 organizations has its own advantages in terms of generalizability. But a key trade-off is that we were not able to consider structural characteristics of the networks beyond one node from the survey respondent. The structural measures were also heavily reliant on the respondents’ perceptions of ties among people in their networks (Krackhardt 1990). Although the limitation stemming from the egocentric survey method was our inability to validate the existence of the reported relationships between alters, previous research suggests that people are able to report accurately their typical social relationships (Hansen 1999; Marsden 1990). Future research should explore alternative methods to capture social network structure, such as the sociometric method and collect the full network structures within a well-defined group.

Future study should also explore the direct relationship between regulatory focus orientation and network structure. For example, a recent study showed that promotion-focused entrepreneurs were more likely to have weekly, business-related contact with members of their networking groups, compared to prevention-focused entrepreneurs (Pollack et al. 2015). This suggests that regulatory focus system could play a significant role in shaping people’s networking behaviors and subsequently the network structures. It is also possible that our analyses failed to expose a relationship between a subjects’ regulatory focus and the density of her network because in the professional context, there are many influences on network structure, such as the organizational chart, that an individual can’t control. It may be that in purely social-related networks, a direct relationship between regulatory focus and density will exist. Future studies should start by examining regulatory focus orientation and its associated networking behaviors, such as individual propensity to connect with others (e.g., Totterdell et al. 2008).

Another limitation is that we did not assess the well-being of the immediate social contacts (alters) of egos, which can play a significant role in shaping the ego’s own well-being. Future research should not only measure the overall network structure, but it should also examine the well-being levels of social contacts and their effects on the ego’s happiness. Emerging evidence suggests that individual well-being as well as individual degrees of psychological loneliness can spread over networks (Cacioppo et al. 2009; Fowler and Christakis 2008), suggesting that people who are surrounded by many people high in well-being may have higher levels of well-being themselves.

Examining the well-being of a person’s network contacts has two important implications for future research that would build on the results of the present studies. First, individual differences in promotion and prevention may moderate the “happiness contagion” effect. To the extent that promotion-focused people are more sensitive to well-being related positive affect and are more likely to be engaged by positive outcome cues (see Higgins 1997), the “happiness contagion” effect may be particularly strong among high promotion-focused people. Second, this effect may be weaker among high prevention-focused people, who are less sensitive to happiness-related cues. To capture the distinguishing characteristics of the prevention system, examining a “serenity contagion” effect that reflects the absence of negative feelings among prevention-focused people when they are able to successfully self-regulate would be a necessary and useful step (see Higgins 1997).

Last but not least, we use general life satisfaction as an indicator of subjective well-being. Future studies should examine more fine-grained indicators. In this regard, emotion is a critical component of the self-regulation processes (Higgins 1997), as well as a component of subjective well-being (Busseri and Sadava 2011; Diener 1984; Lucas et al. 1996; Schimmack 2008). Research on regulatory focus theory has documented distinct patterns of emotional sensitivities (Brendl et al. 1995; Shah and Higgins 2001) and emotional reactions to success and failure (Idson et al. 2000) across promotion-focused and prevention-focused individuals. Promotion-focused people are more likely to be aroused by success than failure; they display cheerfulness and high eagerness after success and dejection and low eagerness after failure. By contrast, prevention-focused people are more likely to be aroused by failure than success; they display quiescence and low vigilance after success and agitation and high vigilance after failure. Drawing on these differences in emotional experiences, a novel direction for future studies would be to examine the effect of social network on emotions and emotion regulation. Will people experience distinct emotions as a function of network density? If a high-density network serves people’s prevention-focused concerns for maintaining trust and the status-quo, a low-density network may disrupt prevention-focused self-regulatory processes. Past research has found that disruptions of the prevention system can lead to feelings of failure that can produce emotional syndromes associated with anxiety disorders (Strauman and Higgins 1987). Would prevention-focused people be more susceptible to anxiety disorders in low-density networks than in high density networks?

## Concluding comment

In sum, structural analysis offers an insightful approach to analyzing social situations, and adding motivational theories to structural analysis can help forge a powerful perspective for understanding the interactions between basic psychological mechanisms and social structures. Rather than reifying the division between those interested in psychological mechanisms and those interested in how network structures affect well-being, we suggest an interdisciplinary model that draws from both psychological and sociological analyses to understand how social networks affect individuals’ subjective well-being.

## Appendix

See Tables 3 and 4

**Table 3:** Regression summary in predicting general life satisfaction (Study 1)

	Model 1	Model 2	Model 3	Model 4	Model 5	Model 6
Age	−0.013 (0.008)	−0.007 (0.007)	−0.007 (0.007)	−0.005 (0.007)	−0.007 (0.007)	−0.006 (0.007)
Female	0.166 (0.133)	−0.006 (0.120)	0.015 (0.120)	0.004 (0.123)	−0.016 (0.123)	−0.017 (0.124)
Non-Caucasian	−0.346** (0.115)	−0.196 (0.104)	−0.150 (0.105)	−0.215* (0.104)	−0.195 (0.107)	−0.202 (0.107)
Finance industry	−0.106 (0.090)	−0.052 (0.081)	−0.043 (0.081)	−0.032 (0.083)	−0.035 (0.083)	−0.042 (0.083)
No. of different sex contact	0.001 (0.012)	0.001 (0.011)	0.001 (0.011)	0.001 (0.011)	0.001 (0.011)	0.002 (0.011)
No. of different race contact	0.007 (0.009)	0.002 (0.008)	0.000 (0.008)	0.004 (0.008)	0.004 (0.008)	0.004 (0.008)
Network size	−0.027 (0.014)	−0.017 (0.013)	−0.014 (0.013)			
No. of strong ties	0.037*** (0.011)	0.020* (0.010)	0.019 (0.010)	0.017* (0.010)	0.008 (0.009)	0.017 (0.011)
Overall density	0.138** (0.045)	0.115** (0.040)	0.127** (0.040)			
Prevention effectiveness		0.133*** (0.039)	0.146*** (0.039)	0.140*** (0.040)	0.125*** (0.040)	0.138*** (0.040)
Promotion effectiveness		0.441*** (0.040)	0.437*** (0.040)	0.411*** (0.041)	0.439** (0.041)	0.432*** (0.041)
Density × prevention effectiveness			0.107* (0.047)			
Density × promotion effectiveness			−0.008 (0.043)			
No. of ties outside the organization				−0.015 (0.008)		−0.018 (0.014)
Density outside the organization				0.087* (0.041)		
Out-density × prevention effectiveness				0.086* (0.045)		
Out-density × promotion effectiveness				−0.136** (0.044)		
No. of ties inside the organization					−0.012 (0.008)	−0.005 (0.014)
Density inside the organization					0.092* (0.041)	
In-density × prevention effectiveness					0.029 (0.044)	
In-density × promotion effectiveness					−0.037 (0.038)	
Density cross the organization						0.079 (0.041)
Cross-density × prevention effectiveness						0.101* (0.044)
Cross-density × promotion effectiveness						0.031 (0.047)
Constant	5.320*** (0.392)	5.150*** (0.351)	5.100*** (0.351)	4.962*** (0.345)	4.841*** (0.348)	5.033*** (0.374)
R-squared	0.069	0.256	0.263	0.260	0.247	0.247

Standard errors in parentheses

*** *p* < .001; ** *p* < .01; * *p* < .05

**Table 4:** Regression analyses summary (Study 2)

	Model 1	Model 2	Model 3	Model 4	Model 5	Model 6	Model 7	Model 8	Model 9
	Life satisfaction	Life satisfaction	Life satisfaction	Prevention support	Prevention support	Promotion support	Promotion support	Life satisfaction	Life satisfaction
Female	−0.041 (0.169)	−0.095 (0.151)	−0.067 (0.149)	0.016 (0.086)	0.026 (0.086)	−0.020 (0.120)	−0.037 (0.121)	−0.050 (0.170)	−0.035 (0.151)
Age	−0.043*** (0.012)	−0.048*** (0.011)	−0.046*** (0.011)	−0.004 (0.006)	−0.003 (0.006)	0.013 (0.009)	0.012 (0.009)	−0.047*** (0.012)	−0.048*** (0.011)
British African	−0.581* (0.260)	−0.474* (0.231)	−0.439 (0.228)	−0.131 (0.132)	−0.117 (0.131)	0.375* (0.184)	0.360 (0.184)	−0.613* (0.262)	−0.490* (0.231)
Fulltime employed	−0.030 (0.215)	−0.163 (0.194)	−0.137 (0.191)	0.227* (0.110)	0.240* (0.110)	−0.040 (0.154)	−0.041 (0.154)	−0.098 (0.215)	−0.166 (0.193)
Network size	0.020** (0.008)	0.011 (0.007)	0.010 (0.007)	0.005 (0.004)	0.007 (0.004)	−0.000 (0.006)	0.000 (0.006)		
Network density	0.175* (0.084)	0.140 (0.075)	0.164* (0.074)	0.068 (0.043)	0.078 (0.042)	−0.188** (0.059)	−0.199*** (0.060)		
Prevention effectiveness		0.173* (0.074)	0.152* (0.073)	−0.018 (0.042)	−0.029 (0.042)	0.026 (0.059)	0.027 (0.059)		0.172* (0.074)
Promotion effectiveness		0.552*** (0.074)	0.529*** (0.074)	0.099* (0.042)	0.092* (0.042)	0.206*** (0.056)	0.223*** (0.059)		0.503*** (0.076)
Density × prevention effectiveness			0.175* (0.076)		0.088* (0.044)		−0.012 (0.061)		
Density × promotion effectiveness			−0.188* (−0.093)		−0.062 (0.054)		0.131 (0.075)		
Prevention support								0.307* (0.128)	0.278* (0.109)
Promotion support								0.147 (0.090)	0.042 (0.075)
Prevention effectiveness × prevention support									0.163* (0.073)
Promotion effectiveness × prevention support									−0.004 (0.064)
Prevention effectiveness × promotion support									−0.032 (0.061)
Promotion effectiveness × promotion support									0.229** (0.082)
Constant	5.035*** (0.708)	5.754*** (0.643)	5.660*** (0.633)	3.513*** (0.366)	3.474*** (0.364)	4.796*** (0.512)	4.836*** (0.511)	3.908*** (0.905)	5.943*** (0.615)
R-squared	0.137	0.325	0.352	0.119	0.131	0.119	0.131	0.133	0.353

Standard errors in parentheses

*** *p* < .001; ** *p* < .01; * *p* < .05

## Footnotes

1 Subjective well-being is about how people feel and think about their lives (Diener 1984). Prior research has shown that it includes two components: affective and cognitive (Busseri and Sadava 2011; Diener 1984; Lucas et al. 1996), which are associated with distinct antecedents and consequences. In this paper, we focus on cognitive well-being, which is typically measured as satisfaction with life (Diener et al. 1985).

2 There was no significant interaction effect between strong ties and regulatory foci. We also conducted additional analyses by adding these two interaction terms as control variables in the subsequent analysis. The results remained the same. Because of the effects of the interaction terms were non-significant, we decided to report the version of results without them as controls.

3 In order to test the hypothesis that the standardized beta weights for promotion effectiveness and prevention effectiveness were statistically significantly different from each other, we first estimate their corresponding 95 % of confidence intervals via corrected bootstrap (1000 resamples), promotion: CI [0.3591, 0.5152], prevention CI[0.0697, 0.2232]. As the confidence intervals overlapped by less than 50 %, the beta weights would considered statistically different from each other (*p* < .05, Cumming 2009)

4 In this study, we collected information on tie strength and the demographic background of alters, as in Study 1. As shown in Study 1, adding these variables as controls did not affect our hypothesis testing. In Study 2, when we conducted additional analyses by using the same set of control variables as Study 1, the results remained the same. For the sake of parsimony, we reported the results without those controls.

5 In order to test the hypothesis that the standardized beta weights for promotion effectiveness and prevention effectiveness were statistically significantly different from each other, we first estimate their corresponding 95 % of confidence intervals via corrected bootstrap (1000 resamples), promotion: CI [0.41, 0.70], prevention CI [0.03, 0.32]. As the confidence intervals overlapped by less than 50 %, the beta weights would considered statistically different from each other (*p* < .05, Cumming 2009)

6 We believe it will be very interesting to study the interaction effects of network structures and personality factors on well-being. However, because it is beyond the scope of the current study, we decided not to report the detailed analysis. We will return to this in the General Discussion section when discussing directions for future research.
